# Respiratory mechanics characteristics at the time of barotrauma presentation in patients with critical COVID-19 infection

**DOI:** 10.62675/2965-2774.20240248-en

**Published:** 2024-08-07

**Authors:** Gabriela Vieira Steckert, Sophia Andreola Borba, Gabriela Meirelles Marchese, Fabrício Schultz Medeiros, Tiago Severo Garcia, Marcio Manozzo Boniatti, Iuri Christmann Wawrzeniak

**Affiliations:** 1 Universidade Federal do Rio Grande do Sul Hospital de Clínicas de Porto Alegre Porto Alegre RS Brazil Hospital de Clínicas de Porto Alegre, Universidade Federal do Rio Grande do Sul -Porto Alegre (RS), Brazil.; 2 Hospital Divina Providência Porto Alegre RS Brazil Hospital Divina Providência - Porto Alegre (RS), Brazil.

**Keywords:** Barotrauma, Pulmonary embolism, Pneumothorax: COVID-19, Coronavirus infections, Ventilators, mechanical, Respiration, artificial, Respiratory mechanics

## Abstract

**Objective:**

To evaluate how ventilatory support, the duration of invasive ventilatory support use and lung mechanics are related to barotrauma development in patients who are severely infected with COVID-19 and who are admitted to the intensive care unit and develop pulmonary barotrauma.

**Methods:**

Retrospective cohort study of patients who were severely infected with COVID-19 and who developed pulmonary barotrauma secondary to mechanical ventilation.

**Results:**

This study included 60 patients with lung barotrauma who were divided into two groups: 37 with early barotrauma and 23 with late barotrauma. The early barotrauma group included more individuals who needed noninvasive ventilation (62.2% *versus* 26.1%, p = 0.01). The tidal volume/kg of predicted body weight on the day of barotrauma was measured, and 24 hours later, it was significantly greater in the late barotrauma group than in the early barotrauma group. During the day, barotrauma was accompanied by plateau pressure and driving pressure accompanied by tidal volume, which significantly increased in the late barotrauma group. According to the SAPS 3, patients in the early barotrauma group had more pulmonary thromboembolism and more severe illness. However, the intensive care unit mortality rates did not significantly differ between the two groups (66.7% for early barotrauma *versus* 76.9% for late barotrauma).

**Conclusion:**

We investigated the effect of respiratory mechanics on barotrauma in patients with severe COVID-19 and found that 25% of patients were on nonprotective ventilation parameters when they developed barotrauma. However, 50% of patients were on protective ventilation parameters, suggesting that other nonventilatory factors may contribute to barotrauma.

## INTRODUCTION

The development of positive pressure ventilators is currently considered one of the greatest medical achievements, allowing severely ill patients not only to survive for longer periods but also to become candidates for healing therapies previously inconceivable due to the severity of their disease.^(1)^ Mechanical ventilation (MV) has become one of the most important supportive therapies for acute respiratory distress syndrome (ARDS), with a wide range of complex strategies that balance ventilation support and disease-related lung injuries.^(2)^ However, positive pressure ventilation is not harmless, and pulmonary barotrauma is still a major concern among specialists, given that high levels of lung distending pressure are associated with injury.^(3,4)^

Since 2020, severe coronavirus disease 2019 (COVID-19) infection has emerged as a global health challenge, with more than 750 million diagnosed cases and almost 7 million deaths worldwide.^(5)^ Although patients infected by COVID-19 show considerable respiratory distress, physicians noticed that this subpopulation presented higher rates of spontaneous pneumothorax, pneumomediastinum and subcutaneous emphysema than first expected.^(6–15)^ The incidence of spontaneous pneumomediastinum and pneumothorax may vary between 3% and 10% in COVID-19 patients,^(16–18)^ suggesting that an additional component of lung frailty is associated with COVID-19-related ARDS. In addition, whenever these patients were treated with MV, the number of pulmonary barotrauma patients increased compared to that of patients with other ARDS etiologies. A single-center retrospective study showed that among 116 patients with COVID-19-related ARDS, almost one out of four who required MV developed pneumothorax or pneumomediastinum, and these complications were also associated with an increased mortality risk.^(19)^ The results from a prospective cohort are similar to those of a previous study: COVID-19 patients had a 13.6% incidence of pneumomediastinum or subcutaneous emphysema, whereas non-COVID-19 patients had a 1.9% incidence, which is significantly lower.^(20)^ Therefore, an important factor in this population is that patients must remain on ventilatory support for a long time, which could be the main cause of barotrauma due to the risk of ventilator-associated lung injury (VALI).^(21)^ Another relevant factor is the need for noninvasive ventilation (NIV) support to meet patients’ high demands and the scarcity of invasive resources in many care centers for patients with severe COVID-19 infections.^(19,22,23)^

In the present study, we evaluated patients with severe COVID-19 infections who were admitted to the ICU and developed pulmonary barotrauma, aiming to investigate how ventilatory support, the duration of invasive ventilatory support use and lung mechanics are related to barotrauma development.

## METHODS

### Study design and population

This retrospective cohort study was carried out at a tertiary-level university hospital, which evaluated data from patients who were admitted to the ICU due to severe COVID-19 infections and who developed pulmonary barotrauma secondary to MV. The Ethical Committee of the *Hospital de Clínicas de Porto Alegre* approved this study (project number: 2020-0619/CAAE: 40761220500005327). All procedures were followed in accordance with the ethical standards of the institutional committee and with the Helsinki Declaration of 1975. Since this study involved retrospective research, this analysis waived the need for individual informed consent; however, the data remained confidential, and access to the data was restricted to the authors. Data were extracted from inpatients’ medical electronic records, and patient selection was performed using keywords referring to radiological findings supporting barotrauma (defined as "pneumothorax", "pneumomediastinum" and "subcutaneous emphysema"). The data were collected from 01 March 2020 to 31 March 2021. The eligibility criteria included patients who were older than 18 years, who had COVID-19 infection diagnostics established by polymerase chain reaction (PCR) or antigen methods, who were under MV and who developed any barotrauma type. The following potential confounders were defined as exclusion criteria: previous pneumothorax history, thoracic surgery or pleurodesis, the need for extracorporeal membrane oxygenation, and patients who were already receiving MV when admitted to the hospital. Patients were divided into two groups based on when barotrauma developed after beginning MV: early barotrauma, defined as barotrauma that developed within 7 days after MV started; and late barotrauma, defined as barotrauma that developed after this period.

### Data collection

The following data were extracted: demographic and epidemiologic characteristics (age, sex, body mass index, previous comorbidities, Simplified Acute Physiology Score 3 [SAPS 3]); clinical parameters during hospitalization (C-reactive protein, D-dimers, partial pressure of oxygen/fraction of inspired oxygen [PaO_2_/FiO_2_] ratio, NIV use before intubation, high-flow catheter, prone position, dialysis, length of stay, occurrence of venous thromboembolism or ventilator-associated pneumonia, prone position, and vasopressor necessity). Mechanical ventilation parameters were assessed at three different time points: at the beginning of MV (D1) and after five days (D5), on the day when barotrauma was diagnosed (D0 barotrauma) and after 24 hours (D1 barotrauma). The lung-protective ventilation strategy included limited tidal volumes (4 - 88mL/kg predicted body weight) and inspiratory pressures (plateau pressure, 30cmH_2_O).^(24)^

### Statistical analysis

The categorical variables are presented as relative and absolute frequencies. Continuous variables are reported as the mean ± standard deviation (SD) or median and interquartile range, as appropriate. Both the early and late barotrauma groups were compared according to the data type. Mann–Whitney and Kruskal–Wallis tests were performed for nonparametric variables. To compare categorical variables, the chi-square test was used, except when the expected frequencies in contingency tables were less than 5, for which Fisher's exact test was used. Statistical analysis was performed using *Statistical* Package for the Social Sciences (SPSS: IBM, Chicago, Illinois). A p value < 0.05 was considered to indicate statistical significance.

## RESULTS

**Figure 1:**
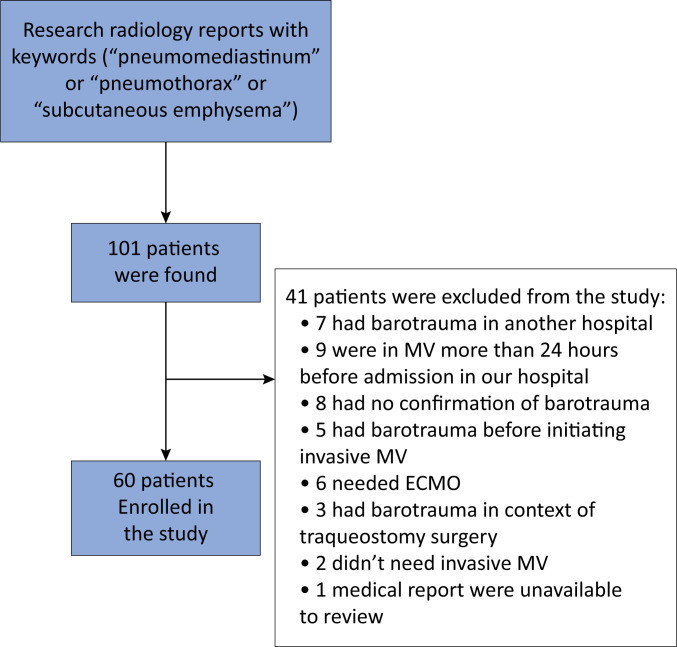
Screening of the patients.

**Figure 2:**
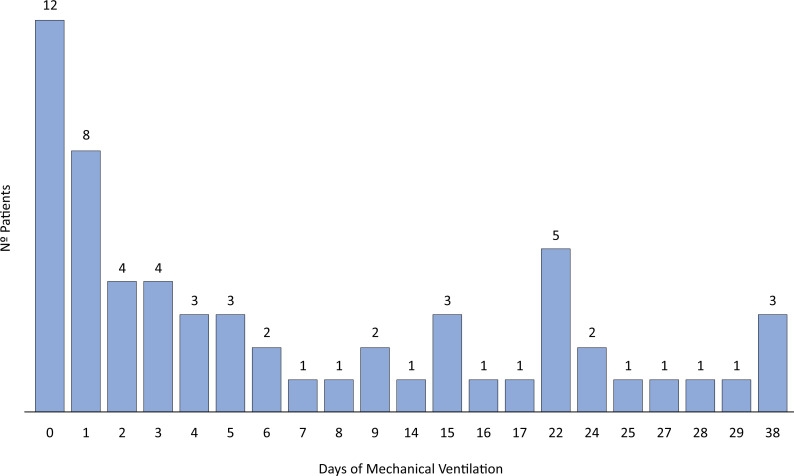
Histogram of the timing of barotrauma in the included patients.

In our first evaluation, 101 individuals were identified according to the inclusion criteria. After the analysis of the exclusion criteria, a total of 60 patients with COVID-19-related ARDS who developed lung barotrauma under MV were enrolled in our study (Figure 1) and divided into two subgroups according to the time at which barotrauma occurred: 37 subjects in the early barotrauma group and 23 in the late barotrauma group. Figure 2 shows a histogram of the timing of barotrauma in the included patients. Table 1 summarizes the patients’ demographic and clinical characteristics. According to the descriptive analysis, the early barotrauma group had more individuals who needed NIV (62.2% *versus* 26.1%; p = 0.01) and more patients with higher illness severity rates according to the SAPS 3 (65 ± 16 *versus* 55 ± 10; p = 0.02).

MV - mechanical ventilation; ECMO - extracorporeal membrane oxygenation.

**Table 1:** Epidemiological characteristics and interventions of intensive care unit patients with barotrauma

			Total (n = 60)	Early barotrauma (n = 37)	Late barotrauma (n = 23)	p value
Epidemiological characteristics						
	Age (years)		60.5 ± 12	60.5 ± 14	60.6 ± 9	0.97
	Sex, masculine		40 (66.7)	24 (64.9)	16 (69.6)	0.78
	SAPS 3		61 ± 15	65 ± 16	55 ± 10	0.02
	Comorbidity					
		Arterial hypertension	39 (65)	27 (73)	12 (52)	0.16
		Diabetic mellitus	18 (30)	9 (24)	9 (39)	0.26
		Chronic kidney disease	10 (17)	8 (22)	2 (9)	0.29
		Ischemic heart disease	6 (10)	5 (14)	1 (4)	0.39
		Obesity	22 (37)	15 (41)	7 (30)	0.58
		Previous lung disease*	2 (3)	2 (5)	0 (0)	0.52
	C-reactive protein		177 ± 92	189 ± 97	159 ± 80	0.31
	D-dimers		3.24 ± 4	3.57 ± 5	2.74 ± 4	0.92
	PaO_2_/FiO_2_ (mmHg)		173 ± 74	182 ± 78	159 ± 66	0.29
	ARDS classification					0.72
		Mild	22 (36.7)	15 (40.5)	7 (30.4)	-
		Moderate	28 (46.7)	16 (43.2)	12 (52.2)	-
		Severe	10 (16.7)	6 (16.2)	4 (17.4)	-
Interventions						
	Corticosteroids time until MV (days)		3.7 ± 4	4,2 ± 4	2.95 ± 3	0.29
	High flow catheter		24 (40)	17 (45.9)	7 (30.4)	0.29
	Noninvasive ventilation		29 (48.3)	23 (62.2)	6 (26.1)	0.01
	Prone position		35 (58.3)	22 (59.5)	13 (56.5)	> 0.99
	Hemodialysis		25 (41.7)	13 (35.1)	12 (52.2)	0.28
	Vasopressor		59 (98.3)	36 (97.3)	23 (100)	> 0.99

SAPS 3 - Simplified Acute Physiology Score 3; PaO_2_ - partial pressure of oxygen; FiO_2_ - fraction of inspired oxygen; ARDS - acute respiratory distress syndrome; MV - mechanical ventilation. Results expressed as the mean ± standard deviation or n (%). Early barotrauma was defined as barotrauma occurring within 7 days after mechanical ventilation started, and late barotrauma was defined as barotrauma occurring 7 days after mechanical ventilation started.

* Asthma or chronic obstructive pulmonary disease. Mann-Whitney test or chi-square test or Fisher's test.

Table 2 presents information related to the MV parameters. An important finding was that the difference in tidal volume/ml of predicted body weight on the day of barotrauma was detected, and 24 hours later, the difference was significantly greater in the late barotrauma group than in the early barotrauma group. In addition, on the day that barotrauma occurred, the plateau pressure, driving pressure (DP) and tidal volume significantly increased in the late barotrauma group.

**Table 2:** Parameters of mechanical ventilation in patients with barotrauma

Variable		Total (n = 60)	Early barotrauma (n = 37)	Late barotrauma (n = 23)	p value
D0 initial MV					
	Vt/PBW (mL/kg)	60	6.6 (6 - 7)	6.5 (6 - 7)	0.72
	PEEP	60	12 (10 - 14)	12 (10 - 14)	0.77
	Plateau pressure	49	28 (26 - 31)	25 (24 - 28)	0.004
	Driving pressure	49	16 (12 - 18)	13 (11 - 14)	0.02
	Cstat (mL/cmH_2_O)	49	27 (23 - 38)	33 (28 - 38)	0.07
	Cstat/PBW (mL/cmH_2_O/kg)	49	0.44 (0.4 - 0.6)	0.53 (0.4 - 0.6)	0.03
	PIP	28	32 (25 - 35)	32 (24 - 35)	0.96
	RR	60	28 (25 - 30)	25 (24 - 30)	0.14
D5 after beginning MV					
	Vt/PBW (mL/kg)	60	6.8 (6 - 8)	6.9 (6 - 9)	0.48
	PEEP	60	10 (8 - 12)	10 (8 - 12)	0.49
	Plateau pressure	37	25 (24 - 28)	25 (22 - 28)	0.54
	Driving pressure	37	15 (13 - 17)	14 (10 - 15)	0.19
	Cstat (mL/cmH_2_O)	37	29(25 - 35)	30 (22 - 37)	0.85
	Cstat/PBW (mL/cmH_2_O/kg)	37	0.46 (0.4 - 0.5)	0.47 (0.4 - 0.6)	0.94
	PIP	35	30 (28 - 33)	25 (23 - 32)	0.03
	RR	60	28 (25 - 31)	26 (20 - 28)	0.06
D0 MV barotrauma					
	Vt/PBW (mL/kg)	60	6.7 (6 - 7)	7.6 (6 - 9)	0.047
	PEEP	60	12 (10 - 14)	10 (8 - 10)	0.002
	Plateau pressure	32	28 (24 - 30)	29 (23 - 32)	0.77
	Driving pressure	32	15 (12 - 16)	16 (14 - 22)	0.24
	Cstat (mL/cmH_2_O)	32	30 (23 - 36)	24 (19 - 32)	0.23
	Cstat/PBW (mL/cmH_2_O/kg)	32	0.45 (0.4 - 0.5)	0.46 (0.3 - 0.5)	0.63
	PIP	28	30 (26 - 33)	32 (28 - 36)	0.27
	RR	60	28 (25 - 30)	26 (22 - 32)	0.72
D1 MV barotrauma					
	Vt/PBW (mL/kg)	60	6.6 (6 - 7)	7.1 (7 - 9)	0.02
	PEEP	60	10 (8 - 12)	10 (8 - 10)	0.049
	Plateau pressure	34	22 (22 - 26)	26 (23 - 35)	0.11
	Driving pressure	34	12 (11 - 15)	16 (14 - 22)	0.01
	Cstat (mL/cmH_2_O)	34	35 (26 - 41)	28 (17 - 34)	0.02
	Cstat/PBW (mL/cmH_2_O/kg)	34	0.53 (0.5 - 0.6)	0.42 (0.3 - 0.5)	0.02
	PIP	33	30 (28 - 33)	32 (26 - 40)	0.25
	RR	60	28 (24 - 30)	27 (24 - 30)	0.57

D - day; MV - mechanical ventilation; Vt - tidal volume; PBW - predicted body weight; PEEP - positive end-expiratory pressure; Cstat - static compliance; PIP - peak pressure; RR - respiratory rate. The data are shown as the median (interquartile range). Early barotrauma was defined as barotrauma occurring within 7 days after the start of mechanical ventilation, and late barotrauma was defined as barotrauma occurring 7 days after the start of mechanical ventilation. Mann–Whitney test.

Table 3 shows the data for each analyzed barotrauma subtype. Chest radiography was the main diagnostic imaging tool for detecting barotrauma (58.1%), followed by thoracic computed tomography (CT) (41.9%). In the early barotrauma group, greater pneumomediastinum and subcutaneous emphysema were detected; however, pneumothorax was the same for both groups, with a greater incidence of intervention in the late barotrauma group.

**Table 3:** Types of barotrauma

	Total (n = 60)	Early barotrauma (n = 37)	Late barotrauma (n=23)	p value
Pneumomediastinum	42 (70)	29 (78.4)	13 (56.5)	0.09
Subcutaneous emphysema	35 (58.3)	25 (67.6)	10 (43.5)	0.1
Pneumothorax	26 (43.3)	14 (37.8)	12 (52.2)	0.28
Airway injury during intubation	5 (8.3)	3 (8.1)	2 (8.7)	>0.99
Barotrauma related to catheter insertion	5 (8.3)	3 (8.1)	2 (8.7)	>0.99
Barotrauma requiring intervention	30 (50)	16 (43.2)	14 (60.9)	0.29

Early barotrauma was defined as barotrauma that occurred within 7 days after mechanical ventilation started, and late barotrauma was defined as barotrauma that occurred 7 days after mechanical ventilation started. The data are shown as n (%). Chi-square test or Fisher's test.

Pulmonary thromboembolism was more common in the early group than in the late group (Table 4). The median duration of hospitalization and MV necessity were significantly greater in the late barotrauma group (28 *versus* 42 days; p = 0.01; and 17 *versus* 39 days; p = 0.001, respectively), although the ICU mortality rates of both groups did not significantly differ (66.7% in the early barotrauma group *versus* 76.9% in the late barotrauma group; p = 0.72).

**Table 4:** Complications and outcomes of intensive care unit patients with barotrauma

Variable		Total (n=60)	Early barotrauma (n=37)	Late barotrauma (n=23)	p value
Complications					
	Pulmonary embolism	15 (25)	13 (35.1)	2 (8.7)	0.03
	VAP	51 (85)	29 (78.4)	22 (95.7)	0.13
	Acute renal failure	44 (73.3)	26 (70.3)	18 (78.3)	0.56
Outcomes					
	MV duration	28 (13 - 39)	17 (10 - 32)	39 (30 - 45)	0.001
	ICU LOS	31 (18 - 43)	24 (12 - 36)	40 (30 - 52)	0.001
	Hospital LOS	36 (24 - 48)	28 (16 - 45)	42 (31 - 59)	0.003
	External transfer	8 (13.3)	5 (13.5)	3 (13)	>0.99
	ICU mortality	36 (60)	23 (62.2)	13 (56.5)	0.79

VAP - ventilator-associated pneumonia; MV - mechanical ventilation; ICU - intensive care unit; LOS - length of stay. The data are shown as the median (interquartile range) or n (%). Early barotrauma was defined as barotrauma that occurred within 7 days after mechanical ventilation started, and late barotrauma was defined as barotrauma that occurred 7 days after mechanical ventilation started. Mann–Whitney test, chi-square test or Fisher's test.

## DISCUSSION

We investigated the effect of respiratory mechanics on barotrauma in patients with severe COVID-19. Nonprotective ventilation at the time of barotrauma presentation may affect the development of barotrauma in patients with severe COVID-19. However, other factors unrelated to respiratory mechanics could contribute to barotrauma in this ARDS subpopulation, although more than 50% of patients were under protective ventilation.

Our results revealed a greater incidence of pulmonary embolism, greater illness severity according to the SAPS 3 score and NIV use, mainly in the early barotrauma group. These findings may demonstrate greater inflammatory and thrombotic states and, consequently, suggest greater lung injury, as demonstrated by other clinical studies on COVID-19 infection.^(25,26)^ At the time of the development of early barotrauma, many patients were under NIV support, while patients with late barotrauma were under noninvasive support less frequently. Other authors have suggested that nonprotective ventilation during NIV could cause lung injury, especially in patients with increased spontaneous respiratory effort generated by a high respiratory drive and excessive transpulmonary pressure swings—patient self-inflicted lung injury (P-SILI).^(17,27–29)^ An inappropriate high respiratory drive can lead dyspneic patients to make vigorous efforts and consequently "fight" the respirator, and poor patient-ventilation interactions, mainly noninvasive support.^(30)^ However, more clinical studies are needed to prove the real effects of P-SILI on lung injury in patients with ARDS and, especially, severe COVID-19 infection. Our results showed that at least 25% of the patients were under nonprotective ventilation at the time they developed barotrauma. This finding suggests a potential role for VALI in the presentation of barotrauma. In the literature, these aspects of nonprotective lung mechanics are well defined to avoid VALI in non-COVID-19 ARDS patients and monitor the deleterious effects of prolonged nonprotective NIV use and, when necessary, to start invasive MV to a better protective ventilatory strategy.^(27,31–33)^ An additional aspect worth further exploration is the observation that a significant portion of patients, comprising at least 50%, were maintained under protective ventilation parameters; this underscores the importance of recognizing that even among patients seemingly under protective ventilation, the risk of barotrauma persists. The question of whether adopting even more "protective" ventilation strategies would prove advantageous remains debated. Conflicting studies suggest that COVID-19 infection itself is a cause of barotrauma.^(14,18,20,34–36)^ Clinicians should be aware of the risk of barotrauma even among patients on protective ventilation.

We evaluated two phases of barotrauma. Such a division is not described in the literature, but we suggest a better evaluation of the difference between potential prolonged MV effects and the hypotheses on such effects.^(4,37)^ Our group showed that patients with early barotrauma had greater tendencies toward pneumomediastinum and subcutaneous emphysema than those with late barotrauma. However, patients in the late barotrauma group tended to have greater pneumothorax, requiring drainage despite data showing equal airway iatrogenic injury in both groups. These results suggest different barotrauma development mechanisms and effects of prolonged MV. However, our study was not designed to confirm these hypotheses. Sekhon et al. showed that the possible early development of pneumomediastinum and subcutaneous emphysema, mainly in patients with COVID-19, could be explained by the Macklin effect with severe impairment of pulmonary mechanics.^(38)^ In late barotrauma and pneumothorax patients, the mechanism underlying the development of lung injury could be the same as that in other patients without COVID-19 with prolonged MV. Ferreira et al. showed that protective ventilatory parameters were associated with better outcomes in critically ill patients with COVID-19.^(39)^ Our study demonstrated the same alterations in the compliance of the respiratory system to MV at the time of barotrauma. However, respiratory system compliance, tidal volume and positive end-expiratory pressure (PEEP) adjustments, as well as factors unrelated to MV, could be involved in the development of barotrauma and pneumothorax. Barotrauma is, in some cases, considered a terminal event, causing failure to recover from lung injury, destruction of the lung parenchyma and pulmonary fibrosis.^(40)^

### Limitations

Our study has some limitations for interpreting the results. First, it was retrospective and single-center, leading to a limited number of patients and consequently restricting our sample size. Second, our sample size was not calculated and was limited to the patients included in the present study. Third, our study failed to assess the diverse behaviors of COVID-19 patients and patient care, such as sedation levels and neuromuscular blocker use. Fourth, the indication criteria for the use of NIV or invasive MV were not evaluated. Fifth, since our study depended on appropriate patients’ electronic registers, some parameters were missing for the final statistical analysis, which can lead to biases. Considering the study design and the lack of a control group, our data cannot imply a causal association between severe COVID-19 infection and the development of barotrauma in patients receiving MV. Sixth, the assessment used in the study cannot indicate that changes in respiratory mechanics are strictly related to the impairment of the lung parenchyma caused by VALI and consequently to the development of barotrauma. Seventh, many patients with COVID-19 infection need catheter insertion, and the risk of barotrauma related to puncturing and intubating procedures is increased because these factors could interfere with the results of the study. However, there was no significant difference between the early and late barotrauma groups (Table 3).

## CONCLUSION

We examined respiratory mechanics during the onset of barotrauma in patients with severe COVID-19 infections. Although 25% of patients were on nonprotective ventilation parameters when they developed barotrauma, indicating a potential role for ventilator-associated lung injury, over 50% of patients were on protective ventilation parameters. This finding suggests that factors other than mechanical ventilation or respiratory system mechanics may contribute to barotrauma. It is important for intensivists to recognize that barotrauma can still occur even when protective ventilation parameters are achieved.
