# Supplementary material for: Respiratory mechanics characteristics at the time of barotrauma presentation in patients with critical COVID-19 infection
Source: Crit Care Sci. 2024 Aug 7;36:e20240248en. doi: 10.62675/2965-2774.20240248-en (PMC11463995; doi:10.62675/2965-2774.20240248-en)
Supplement: Translation

# Translation: Características da mecânica respiratória na apresentação do barotrauma em pacientes com infecção grave pela COVID-19

**DOI:** 10.62675/2965-2774.20240248-pt

**Authors:** Gabriela Vieira Steckert, Sophia Andreola Borba, Gabriela Meirelles Marchese, Fabrício Schultz Medeiros, Tiago Severo Garcia, Marcio Manozzo Boniatti, Iuri Christmann Wawrzeniak

**Affiliations:** 1 Porto Alegre RS Brasil Hospital de Clínicas de Porto Alegre, Universidade Federal do Rio Grande do Sul -Porto Alegre (RS), Brasil.; 2 Porto Alegre RS Brasil Hospital Divina Providência - Porto Alegre (RS), Brasil.

**Keywords:** Barotrauma, Embolia pulmonar, Pneumotórax, COVID-19, Infecções por coronavírus, Ventiladores mecânicos, Respiração artificial, Mecânica respiratória

## Abstract

**Objetivo:**

Avaliar como o suporte ventilatório, o tempo de uso do suporte ventilatório invasivo e a mecânica pulmonar estão relacionados ao desenvolvimento de barotrauma em pacientes com infecção grave pela COVID-19 admitidos na unidade de terapia intensiva.

**Métodos:**

Trata-se de estudo de coorte retrospectivo de pacientes com infecção grave pela COVID-19 que desenvolveram barotrauma pulmonar secundário à ventilação mecânica.

**Resultados:**

Este estudo incluiu 60 pacientes com barotrauma pulmonar divididos em dois grupos: 37 com barotrauma precoce e 23 com barotrauma tardio. O grupo com barotrauma precoce incluiu mais indivíduos que precisaram de ventilação não invasiva (62,2% *versus* 26,1%, p = 0,01). O volume corrente/kg de peso corporal previsto no dia do barotrauma foi medido e, 24 horas depois, foi significativamente maior no grupo com barotrauma tardio do que no grupo com barotrauma precoce. Durante o dia, o barotrauma foi acompanhado pela pressão de platô e pela pressão de distensão acompanhada do volume corrente, o qual aumentou significativamente no grupo com barotrauma tardio. De acordo com o SAPS 3, os pacientes do grupo com barotrauma precoce apresentaram mais tromboembolia pulmonar e doença mais grave. Entretanto, as taxas de mortalidade na unidade de terapia intensiva não diferiram significativamente entre os dois grupos (66,7% para barotrauma precoce *versus* 76,9% para barotrauma tardio).

**Conclusão:**

Investigamos o efeito da mecânica respiratória no barotrauma em pacientes com COVID-19 graves e descobrimos que um quarto dos pacientes estava em parâmetros de ventilação não protetores quando desenvolveram barotrauma. No entanto, 50% dos pacientes estavam em parâmetros de ventilação protetora, sugerindo que outros fatores não ventilatórios podem contribuir para o barotrauma.

## INTRODUÇÃO

O surgimento dos ventiladores de pressão positiva é atualmente considerado uma das maiores conquistas médicas, permitindo que pacientes graves não apenas sobrevivam por períodos mais longos, mas também se tornem candidatos a terapias de cura antes inconcebíveis, devido à gravidade de sua doença.^(1)^ A ventilação mecânica (VM) tornou-se uma das terapias de suporte mais importantes da síndrome do desconforto respiratório agudo (SDRA), dentre uma ampla gama de estratégias complexas que equilibram o suporte ventilatório e as lesões pulmonares relacionadas à doença.^(2)^ No entanto, a ventilação com pressão positiva não é inofensiva, e o barotrauma pulmonar ainda é uma grande preocupação entre os especialistas, uma vez que altos níveis de pressão de distensão pulmonar estão associados a lesões.^(3,4)^

Desde 2020, a infecção grave pela doença pelo coronavírus 2019 (COVID-19) surgiu como um desafio global de saúde, com mais de 750 milhões de casos diagnosticados e quase 7 milhões de mortes em todo o mundo.^(5)^ Embora os pacientes infectados pela COVID-19 apresentem considerável desconforto respiratório, os médicos notaram que essa subpopulação apresentava taxas mais altas de pneumotórax espontâneo, pneumomediastino e enfisema subcutâneo do que o esperado inicialmente.^(6–15)^ A incidência de pneumomediastino e pneumotórax espontâneos pode variar entre 3 e 10% em pacientes com COVID-19,^(16–18)^ o que sugere que um componente a mais de fragilidade pulmonar está associado à SDRA relacionada à COVID-19. Além disso, sempre que esses pacientes foram tratados com VM, o número de pacientes com barotrauma pulmonar aumentou em comparação com o de pacientes com outras etiologias de SDRA. Estudo retrospectivo em um único centro mostrou que, entre 116 pacientes com SDRA relacionada à COVID-19, quase um em cada quatro que necessitaram de VM desenvolveu pneumotórax ou pneumomediastino, e essas complicações também foram associadas a um maior risco de mortalidade.^(19)^ Os resultados de uma coorte prospectiva são semelhantes aos de um estudo anterior, em que os pacientes com COVID-19 tiveram incidência de 13,6% de pneumomediastino ou enfisema subcutâneo, enquanto os pacientes sem COVID-19 tiveram incidência de 1,9%, o que é significativamente menor.^(20)^ Assim, um fator importante nessa população é que os pacientes devem permanecer em suporte ventilatório por um longo período, o que pode ser a principal causa de barotrauma devido ao risco de lesão pulmonar associada à VM (LPAV).^(21)^ Outro fator relevante é a necessidade de suporte de ventilação não invasiva (VNI) para atender às altas demandas dos pacientes e a escassez de recursos invasivos em muitos centros de assistência médica para pacientes com infecções graves pela COVID-19.^(19,22,23)^

No presente estudo, avaliamos pacientes com infecções graves pela COVID-19 que foram internados na UTI e desenvolveram barotrauma pulmonar, com o objetivo de investigar como o suporte ventilatório, o tempo de uso do suporte ventilatório invasivo e a mecânica pulmonar estão relacionados ao desenvolvimento do barotrauma.

## MÉTODOS

### Desenho do estudo e população

Trata-se de estudo de coorte retrospectivo realizado em um hospital universitário de nível terciário, que avaliou dados de pacientes internados na UTI devido a infecções graves pela COVID-19 e que desenvolveram barotrauma pulmonar secundário à VM. O Comitê de Ética do Hospital de Clínicas de Porto Alegre aprovou este estudo (número do projeto: 2020-0619/CAAE: 40761220500005327). Todos os procedimentos observaram os padrões éticos do comitê institucional e a Declaração de Helsinque de 1975. Como este estudo envolveu pesquisa retrospectiva, essa análise dispensou a necessidade de Termo de Consentimento Livre e Esclarecido individual; no entanto, os dados permaneceram confidenciais, e o acesso aos dados foi restrito aos autores. Os dados foram extraídos dos prontuários eletrônicos médicos dos pacientes internados, e a seleção dos pacientes foi realizada usando descritores referentes a achados radiológicos que sustentam o barotrauma (definido como "pneumotórax", "pneumomediastino" e "enfisema subcutâneo"). Os dados foram coletados de 1° de março de 2020 a 31 de março de 2021. Os critérios de elegibilidade incluíram pacientes com mais de 18 anos, com diagnóstico de infecção pela COVID-19 estabelecido por reação em cadeia da polimerase (PCR) ou métodos de antígeno, que estavam sob VM e que desenvolveram qualquer tipo de barotrauma. Os seguintes fatores de confusão em potencial foram definidos como critérios de exclusão: histórico de pneumotórax anterior, cirurgia torácica ou pleurodese, necessidade de oxigenação por membrana extracorpórea e pacientes que já estavam recebendo VM quando foram admitidos no hospital. Os pacientes foram divididos em dois grupos, conforme o momento em que o barotrauma ocorreu após o início da VM: barotrauma precoce, definido como o que ocorreu em até 7 dias após o início da VM; e tardio, definido como o barotrauma que ocorreu após esse período.

### Coleta de dados

Extraíram-se os seguintes dados: características demográficas e epidemiológicas (idade, sexo, índice de massa corporal, comorbidades anteriores, *Simplified Acute Physiology Score 3* [SAPS 3]); parâmetros clínicos durante a hospitalização (proteína C-reativa, dímeros D, relação entre pressão parcial de oxigênio e fração inspirada de oxigênio [PaO_2_/FiO_2_], uso de VNI antes da intubação, cateter de alto fluxo, posição em decúbito ventral, diálise, tempo de internação, ocorrência de tromboembolia venoso ou pneumonia associada ao ventilador, posição em decúbito ventral e necessidade de vasopressor). Os parâmetros da VM foram avaliados em três momentos diferentes: no início da VM (D1) e após 5 dias (D5), no dia em que o barotrauma foi diagnosticado (barotrauma D0) e após 24 horas (barotrauma D1). A estratégia de ventilação protetora do pulmão incluiu volumes correntes limitados (4 - 88mL/kg de peso corporal previsto) e pressões inspiratórias (pressão de platô, 30cmH_2_O).^(24)^

### Análise estatística

As variáveis categóricas são apresentadas como frequências relativas e absolutas. As variáveis contínuas são relatadas como média ± desvio-padrão (DP) ou mediana e intervalo interquartil, segundo o caso. Os grupos de barotrauma precoce e tardio foram comparados segundo o tipo de dados. O teste de Mann-Whitney e o teste de Kruskal-Wallis foram realizados para variáveis não paramétricas. Na comparação de variáveis categóricas, foi usado o teste do qui-quadrado de Pearson, salvo quando as frequências esperadas nas tabelas de contingência eram menores que 5, para as quais utilizou-se o teste exato de Fisher. A análise estatística foi realizada com o *Statistical Package for the Social Sciences* (SPSS; IBM, Chicago, Illinois). Considerou-se valor de p < 0,05 para indicar significância estatística.

## RESULTADOS

Em nossa primeira avaliação, 101 indivíduos foram identificados segundo os critérios de inclusão. Após a análise dos critérios de exclusão, 60 pacientes com SDRA relacionada à COVID-19 que desenvolveram barotrauma pulmonar sob VM foram incluídos em nosso estudo (Figura 1) e divididos em dois subgrupos conforme o momento em que ocorreu o barotrauma: 37 indivíduos no grupo com barotrauma precoce e 23 no grupo com barotrauma tardio. A figura 2 mostra um histograma do momento do barotrauma nos pacientes incluídos. A tabela 1 resume as características demográficas e clínicas dos pacientes. Na análise descritiva, o grupo com barotrauma precoce teve mais indivíduos que precisaram de VNI (62,2% *versus* 26,1%; p = 0,01) e mais pacientes com taxas mais altas de gravidade da doença de acordo com o SAPS 3 (65 ± 16 *versus* 55 ± 10; p = 0,02).

**Figura 1:** Triagem dos pacientes.

VM - ventilação mecânica; ECMO - oxigenação por membrana extracorpórea.

**Figura 2:** Histograma do momento do barotrauma nos pacientes incluídos.

**Tabela 1:** Características epidemiológicas e intervenções de pacientes de unidades de terapia intensiva com barotrauma

			Total (n = 60)	Barotrauma precoce (n = 37)	Barotrauma tardio (n = 23)	Valor de p
Características epidemiológicas						
	Idade (anos)		60,5 ± 12	60,5 ± 14	60,6 ± 9	0,97
	Sexo masculino		40 (66,7)	24 (64,9)	16 (69,6)	0,78
	SAPS 3		61 ± 15	65 ± 16	55 ± 10	0,02
	Comorbidade					
		Hipertensão arterial	39 (65)	27 (73)	12 (52)	0,16
		*Diabetes mellitus*	18 (30)	9 (24)	9 (39)	0,26
		Doença renal crônica	10 (17)	8 (22)	2 (9)	0,29
		Doença isquêmica do coração	6 (10)	5 (14)	1 (4)	0,39
		Obesidade	22 (37)	15 (41)	7 (30)	0,58
		Doença pulmonar prévia*	2 (3)	2 (5)	0 (0)	0,52
	Proteína C-reativa		177 ± 92	189 ± 97	159 ± 80	0,31
	Dímeros D		3,24 ± 4	3,57 ± 5	2,74 ± 4	0,92
	PaO_2_/FiO_2_ (mmHg)		173 ± 74	182 ± 78	159 ± 66	0,29
	Classificação da SDRA					0,72
		Leve	22 (36,7)	15 (40,5)	7 (30,4)	-
		Moderada	28 (46,7)	16 (43,2)	12 (52,2)	-
		Grave	10 (16,7)	6 (16,2)	4 (17,4)	-
Intervenções						
	Tempo de uso de corticosteroides até a VM (dias)		3,7 ± 4	4,2 ± 4	2,95 ± 3	0,29
	Cateter de alto fluxo		24 (40)	17 (45,9)	7 (30,4)	0,29
	Ventilação não invasiva		29 (48,3)	23 (62,2)	6 (26,1)	0,01
	Posição em decúbito ventral		35 (58,3)	22 (59,5)	13 (56,5)	> 0,99
	Hemodiálise		25 (41,7)	13 (35,1)	12 (52,2)	0,28
	Vasopressor		59 (98,3)	36 (97,3)	23 (100)	> 0,99

SAPS 3 - *Simplified Acute Physiology Score 3*; PaO_2_ - pressão parcial de oxigênio; FiO_2_ - fração inspirada de oxigênio; SDRA - síndrome do desconforto respiratório agudo; VM - ventilação mecânica. Resultados expressos como média ± desvio-padrão ou n (%). O barotrauma precoce foi definido como o que ocorreu dentro de 7 dias após o início da ventilação mecânica, e o tardio foi definido como o barotrauma que ocorreu 7 dias após o início da ventilação mecânica.

* Asma ou doença pulmonar obstrutiva crônica. Teste de Mann-Whitney ou teste do qui-quadrado de Pearson ou teste de Fisher.

A tabela 2 apresenta informações relacionadas aos parâmetros de VM. Um achado importante refere-se à diferença detectada no volume corrente/mL do peso corporal previsto no dia do barotrauma, e, 24 horas depois, a diferença foi significativamente maior no grupo com barotrauma tardio do que no grupo com barotrauma precoce. Além disso, no dia em que ocorreu o barotrauma, a pressão de platô, a pressão de distensão e o volume corrente aumentaram significativamente no grupo com barotrauma tardio.

**Tabela 2:** Parâmetros de ventilação mecânica em pacientes com barotrauma

Variável		Total (n = 60)	Barotrauma precoce (n = 37)	Barotrauma tardio (n = 23)	Valor de p
D0 VM inicial					
	VC/PCP (mL/kg)	60	6,6 (6 - 7)	6,5 (6 - 7)	0,72
	PEEP	60	12 (10 - 14)	12 (10 - 14)	0,77
	Pressão de platô	49	28 (26 - 31)	25 (24 - 28)	0,004
	Pressão de distensão	49	16 (12 - 18)	13 (11 - 14)	0,02
	Cstat (mL/cmH_2_O)	49	27 (23 - 38)	33 (28 - 38)	0,07
	Cstat/PCP (mL/cmH_2_O/kg)	49	0,44 (0,4 - 0,6)	0,53 (0,4 - 0,6)	0,03
	PIP	28	32 (25 - 35)	32 (24 - 35)	0,96
	FR	60	28 (25 - 30)	25 (24 - 30)	0,14
D5 após o início da VM					
	VC/PCP (mL/kg)	60	6,8 (6 - 8)	6,9 (6 - 9)	0,48
	PEEP	60	10 (8 - 12)	10 (8 - 12)	0,49
	Pressão de platô	37	25 (24 - 28)	25 (22 - 28)	0,54
	Pressão de distensão	37	15 (13 - 17)	14 (10 - 15)	0,19
	Cstat (mL/cmH_2_O)	37	29(25 - 35)	30 (22 - 37)	0,85
	Cstat/PCP (mL/cmH_2_O/kg)	37	0,46 (0,4 - 0,5)	0,47 (0,4 - 0,6)	0,94
	PIP	35	30 (28 - 33)	25 (23 - 32)	0,03
	FR	60	28 (25 - 31)	26 (20 - 28)	0,06
D0 Barotrauma de VM					
	VC/PCP (mL/kg)	60	6,7 (6 - 7)	7,6 (6 - 9)	0,047
	PEEP	60	12 (10 - 14)	10 (8 - 10)	0,002
	Pressão de platô	32	28 (24 - 30)	29 (23 - 32)	0,77
	Pressão de distensão	32	15 (12 - 16)	16 (14 - 22)	0,24
	Cstat (mL/cmH_2_O)	32	30 (23 - 36)	24 (19 - 32)	0,23
	Cstat/PCP (mL/cmH_2_O/kg)	32	0,45 (0,4 - 0,5)	0,46 (0,3 - 0,5)	0,63
	PIP	28	30 (26 - 33)	32 (28 - 36)	0,27
	FR	60	28 (25 - 30)	26 (22 - 32)	0,72
D1 Barotrauma de VM					
	VC/PCP (mL/kg)	60	6,6 (6 - 7)	7,1 (7 - 9)	0,02
	PEEP	60	10 (8 - 12)	10 (8 - 10)	0,049
	Pressão de platô	34	22 (22 - 26)	26 (23 - 35)	0,11
	Pressão de distensão	34	12 (11 - 15)	16 (14 - 22)	0,01
	Cstat (mL/cmH_2_O)	34	35 (26 - 41)	28 (17 - 34)	0,02
	Cstat/PCP (mL/cmH_2_O/kg)	34	0,53 (0,5 - 0,6)	0,42 (0,3 - 0,5)	0,02
	PIP	33	30 (28 - 33)	32 (26 - 40)	0,25
	FR	60	28 (24 - 30)	27 (24 - 30)	0,57

D - dia; VM - ventilação mecânica; VC - volume corrente; PCP - peso corporal previsto; PEEP - pressão expiratória positiva final; Cstat - complacência estática; PIP - pressão de pico; FR - frequência respiratória. Resultados expressos como mediana (intervalo interquartil). O barotrauma precoce foi definido como o que ocorreu dentro de 7 dias após o início da ventilação mecânica, e o tardio foi definido como o barotrauma que ocorreu 7 dias após o início da ventilação mecânica. Teste de Mann-Whitney.

A tabela 3 mostra os dados de cada subtipo de barotrauma analisado. A radiografia de tórax foi a principal ferramenta de diagnóstico por imagem para detectar o barotrauma (58,1%), seguida da tomografia computadorizada (TC) torácica (41,9%). No grupo com barotrauma precoce, foram detectados mais pneumomediastino e enfisema subcutâneo; no entanto, o pneumotórax foi o mesmo em ambos os grupos, com maior incidência de intervenção no grupo com barotrauma tardio.

**Tabela 3:** Tipos de barotrauma

	Total (n = 60)	Barotrauma precoce (n = 37)	Barotrauma tardio (n = 23)	Valor de p
Pneumomediastino	42 (70)	29 (78,4)	13 (56,5)	0,09
Enfisema subcutâneo	35 (58,3)	25 (67,6)	10 (43,5)	0,1
Pneumotórax	26 (43,3)	14 (37,8)	12 (52,2)	0,28
Lesão das vias aéreas durante a intubação	5 (8,3)	3 (8,1)	2 (8,7)	> 0,99
Barotrauma relacionado à inserção de cateter	5 (8,3)	3 (8,1)	2 (8,7)	> 0,99
Barotrauma envolvendo intervenção	30 (50)	16 (43,2)	14 (60,9)	0,29

O barotrauma precoce foi definido como o que ocorreu dentro de 7 dias após o início da ventilação mecânica, e o tardio foi definido como o barotrauma que ocorreu 7 dias após o início da ventilação mecânica. Resultados expressos como n (%). Teste do qui-quadrado de Pearson ou teste de Fisher.

O tromboembolia pulmonar foi mais comum no grupo precoce do que no grupo tardio (Tabela 4). A mediana do tempo de hospitalização e a necessidade de VM foram significativamente maiores no grupo com barotrauma tardio (28 *versus* 42 dias; p = 0,01; e 17 *versus* 39 dias; p = 0,001, respectivamente), embora as taxas de mortalidade na UTI de ambos os grupos não tenham sido significativamente diferentes (66,7% no grupo com barotrauma precoce *versus* 76,9% no grupo com barotrauma tardio; p = 0,72).

**Tabela 4:** Complicações e desfechos de pacientes com barotrauma na unidade de terapia intensiva

Variável		Total (n = 60)	Barotrauma precoce (n = 37)	Barotrauma tardio (n = 23)	Valor de p
Complicações					
	Embolia pulmonar	15 (25)	13 (35,1)	2 (8,7)	0,03
	PAV	51 (85)	29 (78,4)	22 (95,7)	0,13
	Insuficiência renal aguda	44 (73,3)	26 (70,3)	18 (78,3)	0,56
Desfechos					
	Duração da VM	28 (13 - 39)	17 (10 - 32)	39 (30 - 45)	0,001
	Tempo de internação na UTI	31 (18 - 43)	24 (12 - 36)	40 (30 - 52)	0,001
	Tempo de internação hospitalar	36 (24 - 48)	28 (16 - 45)	42 (31 - 59)	0,003
	Transferência externa	8 (13,3)	5 (13,5)	3 (13)	>0,99
	Mortalidade na UTI	36 (60)	23 (62,2)	13 (56,5)	0,79

PAV - pneumonia associada ao ventilador; VM - ventilação mecânica; UTI - unidade de terapia intensiva. Resultados expressos como mediana (intervalo interquartil) ou n (%). O barotrauma precoce foi definido como o que ocorreu dentro de 7 dias após o início da ventilação mecânica, e o tardio foi definido como o barotrauma que ocorreu 7 dias após o início da ventilação mecânica. Teste de Mann-Whitney, teste do qui-quadrado de Pearson ou teste de Fisher.

## DISCUSSÃO

Investigamos o efeito da mecânica respiratória no barotrauma em pacientes com COVID-19 graves. A ventilação não protetora no momento da apresentação do barotrauma pode afetar o desenvolvimento do barotrauma em pacientes com COVID-19 graves. No entanto, outros fatores não relacionados à mecânica respiratória podem contribuir para o barotrauma nessa subpopulação de SDRA, embora mais de 50% dos pacientes estivessem sob ventilação protetora.

Nossos resultados revelaram maior incidência de embolia pulmonar, maior gravidade da doença conforme a pontuação SAPS 3 e uso de VNI, principalmente no grupo com barotrauma precoce. Esses achados podem demonstrar maiores estados inflamatórios e trombóticos e, consequentemente, sugerir maior lesão pulmonar, conforme demonstrado por outros estudos clínicos sobre a infecção pela COVID-19.^(25,26)^ No momento do desenvolvimento do barotrauma precoce, muitos pacientes estavam sob suporte de VNI, enquanto os pacientes com barotrauma tardio estavam sob suporte não invasivo com menos frequência. Outros autores sugeriram que a ventilação não protetora durante a VNI poderia causar lesão pulmonar, especialmente em pacientes com aumento do esforço respiratório espontâneo gerado por um alto estímulo respiratório e oscilações excessivas da pressão transpulmonar - lesão pulmonar autoinfligida pelo paciente (P-SILI).^(17,27–29)^ Um estímulo respiratório elevado inadequado pode induzir os pacientes dispneicos a fazer esforços vigorosos e, consequentemente, "lutar" contra o respirador, além de haver interações inadequadas entre paciente e ventilação, principalmente suporte não invasivo.^(30)^ No entanto, outros estudos clínicos são necessários para comprovar os efeitos reais da P-SILI na lesão pulmonar em pacientes com SDRA e, principalmente, infecção grave pela COVID-19. Nossos resultados mostraram que pelo menos 25% dos pacientes estavam sob ventilação não protetora no momento em que desenvolveram barotrauma. Esse achado sugere um possível papel do LPAV na apresentação do barotrauma. Na literatura, esses aspectos da mecânica pulmonar não protetora são bem definidos para evitar LPAV em pacientes sem COVID-19 com SDRA e monitorar os efeitos deletérios do uso prolongado de VNI não protetora e, quando necessário, iniciar a VM invasiva para uma melhor estratégia ventilatória protetora.^(27,31–33)^ Um aspecto adicional que merece ser explorado é a observação de que parcela significativa dos pacientes, pelo menos 50%, foi mantida sob parâmetros de ventilação protetora; isso ressalta a importância de reconhecer que, mesmo entre os pacientes aparentemente sob ventilação protetora, o risco de barotrauma persiste. Discute-se se a adoção de estratégias de ventilação ainda mais "protetoras" seria vantajosa. Estudos conflitantes sugerem que a infecção pela COVID-19 em si é uma causa de barotrauma.^(14,18,20,34–36)^ Os médicos devem estar cientes do risco de barotrauma mesmo entre pacientes em ventilação protetora.

Avaliamos duas fases do barotrauma. Essa divisão não é descrita na literatura, mas sugerimos uma melhor avaliação da diferença entre os potenciais efeitos prolongados da VM e as hipóteses sobre esses efeitos.^(4,37)^ Nosso grupo mostrou que os pacientes com barotrauma precoce tinham maior tendência a pneumomediastino e enfisema subcutâneo do que aqueles com barotrauma tardio. No entanto, os pacientes do grupo com barotrauma tardio tenderam a ter mais pneumotórax, exigindo drenagem, apesar de os dados mostrarem lesões iatrogênicas nas vias aéreas iguais em ambos os grupos. Esses resultados sugerem diferentes mecanismos de desenvolvimento de barotrauma e efeitos da VM prolongada. No entanto, nosso estudo não foi projetado para confirmar essas hipóteses. Sekhon et al. mostraram que o possível desenvolvimento precoce de pneumomediastino e enfisema subcutâneo, principalmente em pacientes com COVID-19, poderia ser explicado pelo efeito Macklin com grave comprometimento da mecânica pulmonar.^(38)^ Em pacientes com barotrauma e pneumotórax tardios, o mecanismo subjacente ao desenvolvimento de lesão pulmonar poderia ser o mesmo de outros pacientes sem COVID-19 com VM prolongada. Ferreira et al. mostraram que os parâmetros ventilatórios protetores estavam associados a melhores desfechos em pacientes com COVID-19 graves.^(39)^ Nosso estudo demonstrou as mesmas alterações na complacência do sistema respiratório à VM no momento do barotrauma. Entretanto, a complacência do sistema respiratório, o volume corrente e os ajustes da pressão expiratória positiva final (PEEP), bem como fatores não relacionados à VM, podem estar envolvidos no desenvolvimento de barotrauma e pneumotórax. O barotrauma é, em alguns casos, considerado um evento terminal, causando falha na recuperação da lesão pulmonar, destruição do parênquima pulmonar e fibrose pulmonar.^(40)^

### Limitações

Nosso estudo tem algumas limitações para a interpretação dos resultados. Primeiramente, trata-se de estudo retrospectivo e feito em um único centro, o que levou a um número limitado de pacientes e, consequentemente, restringiu o tamanho da nossa amostra. Em segundo lugar, o tamanho da nossa amostra não foi calculado e foi limitado aos pacientes incluídos no presente estudo. Terceiro, nosso estudo não avaliou os diversos comportamentos dos pacientes com COVID-19 e a assistência ao paciente, como os níveis de sedação e o uso de bloqueadores neuromusculares. Em quarto lugar, os critérios de indicação para o uso de VNI ou VM invasiva não foram avaliados. Quinto, como nosso estudo dependia de registros eletrônicos apropriados dos pacientes, alguns parâmetros estavam faltando para a análise estatística final, o que pode levar a vieses. Considerando o desenho do estudo e a falta de um grupo controle, nossos dados não podem implicar uma associação causal entre a infecção grave pela COVID-19 e o desenvolvimento de barotrauma em pacientes que recebem VM. Em sexto lugar, a avaliação usada no estudo não permite indicar que as alterações na mecânica respiratória estejam estritamente relacionadas ao comprometimento do parênquima pulmonar causado pela LPAV e, consequentemente, ao desenvolvimento de barotrauma. Em sétimo lugar, muitos pacientes com infecção pela COVID-19 precisam de inserção de cateter, e o risco de barotrauma relacionado aos procedimentos de punção e intubação é maior, pois esses fatores podem interferir nos resultados do estudo. No entanto, não houve diferença significativa entre os grupos de barotrauma precoce e tardio (Tabela 3).

## CONCLUSÃO

Examinamos a mecânica respiratória durante o início do barotrauma em pacientes com infecções graves pela COVID-19. Embora 25% dos pacientes estivessem em parâmetros de ventilação não protetora quando desenvolveram barotrauma, indicando um possível papel para a lesão pulmonar associada à ventilação, mais de 50% dos pacientes estavam em parâmetros de ventilação protetora. Esse achado sugere que outros fatores, além da ventilação mecânica ou da mecânica do sistema respiratório, podem contribuir para o barotrauma. É importante que os intensivistas reconheçam que o barotrauma ainda pode ocorrer mesmo quando forem alcançados parâmetros de ventilação protetora.
